# Efficacy of idarucizumab, prothrombin complex concentrate (PCC) and activated PCC to reverse the anticoagulatory potential of dabigatran in a porcine polytrauma model

**DOI:** 10.1186/cc14431

**Published:** 2015-03-16

**Authors:** M Honickel, T Braunschweig, J Van Ryn, R Rossaint, O Grottke

**Affiliations:** 1 RWTH Aachen University Hospital, Aachen, Germany; 2 CardioMetabolic Diseases Research, Boehringer Ingelheim GmbH & Co KG, Biberach, Germany

## Introduction

The anticoagulant effect of dabigatran can be reversed with idarucizumab or PCCs in porcine blood *in vitro *[1]. However, the impact on clinical parameters such as blood loss is not known. Thus, this study assessed the efficacy of idarucizumab in comparison with PCC and aPCC in dabigatran-anticoagulated swine following polytrauma on clinically relevant endpoints.

## Methods

After ethical approval, 28 male pigs were administered dabigatran etexilate (30 mg/kg twice daily p.o.) for 3 days. Dabigatran was administered intravenously in anaesthetised animals on day 4 to achieve consistent high concentrations. Animals were randomised to receive idarucizumab (60 mg/kg, *n *= 7), PCC (50 U/kg; *n *= 7), aPCC (50 U/kg; *n *= 7) or placebo (*n *= 7). Intervention started 12 minutes after bilateral femur fractures and a standardised blunt liver injury. The primary endpoint was blood loss (observation period 300 minutes). Further, histopathology, haemodynamics and several coagulation variables were also assessed. Data were analysed by repeated-measures ANOVA (mean ± SD).

## Results

Dabigatran levels were comparable between groups (571 ± 174 ng/ml) and resulted in altered coagulation variables. Blood loss was comparable 12 minutes post trauma between groups (801 ± 49 ml) and increased to 3,816 ± 236 ml in anticoagulated control animals post injury. Idarucizumab treatment reduced total blood loss to 1,086 ± 55 ml (*P *< 0.005 vs. all), aPCC to 1,639 ± 104 ml (*P *< 0.05 vs. control) and PCC to 1,797 ± 80 ml (*P *< 0.05 vs. control) after 5 hours. All animals in the intervention groups survived, whereas control animals died within the observation period (mean survival: 89 minutes, range: 62 to 145 minutes). In histopathology no signs of thromboembolic events were present. Altered coagulation variables returned to baseline levels after idarucizumab application and were also significantly, although inconsistently and to a lesser extent, ameliorated following PCCs.

## Conclusion

All medical interventions were associated with reduced blood loss and increased survival. However, idarucizumab, a specific antidote to dabigatran, reduced total blood loss more prominently and normalised coagulation parameters to a greater degree as compared with either PCC or aPCC.
